# 92090 Novel use of REDCap to develop a Crisis Management Decision-Support Portal at an Academic Medical Center

**DOI:** 10.1017/cts.2021.492

**Published:** 2021-03-30

**Authors:** Barbara Tafuto, Megha Master, Doreen Waldron Lechner

**Affiliations:** Department of Health Informatics, School of Health Professions, Rutgers, The State University of New Jersey, Newark, NJ

## Abstract

ABSTRACT IMPACT: Implementation of digital and mobile applications in clinical research crisis response protocols can mitigate their impact on clinical research. OBJECTIVES/GOALS: Crisis management is fundamental to ensuring the protection of human subjects during a clinical trial. Mobile apps have the capacity to allow for quick access to crisis response plans and can serve to mitigate their impact on clinical research. Electronic data capture tools can be used to create digital applications for such a response. METHODS/STUDY POPULATION: The Clinical Research Center at Rutgers University transferred a paper-based crisis management response plan to a digital mobile application format using Vanderbuilt’s Research Electronic Data Capture Tool (REDCap). REDCap’s branching logic allowed for programming a decision support functionality to guide users through 3 specific crises. (1) threatening or aggressive study subject behavior; (2) clinical research center break in or theft; and (3) adverse event observed for ongoing or closed clinical research study. Applicable U.S. Code of Federal Regulations, IRB guidelines, institutional policies and procedures were also used as a component of the mobile development. RESULTS/ANTICIPATED RESULTS: The Crisis Management Decision-Support Portal within the Clinical Research Center has an interactive structure. The use of branching logic demonstrates the ability for users to answer each question and be guided to appropriate responses specific to selected criteria related to each event. The tool contains a self-correct function by providing a reset option after answering each section. The portal itself can be accessed using any computer or cellphone with an internet connection. It provides the users with appropriate criteria to determine to exact communication and management protocols for reporting for the crisis event they are witnessing. As users access the portal, usage data can be collected, tracked, and stored. DISCUSSION/SIGNIFICANCE OF FINDINGS: The REDCap platform offers the opportunity to digitize crisis management protocols that ensure professionals have easy access to appropriate responses to crises at the center. The use a digital application rather than paper allows for a CTSA sponsored hub to mitigate damage, simply build new protocols, modify existing protocols, and track usage.

